# Pathology

**Published:** 1879-01

**Authors:** 


### PATHOLOGY.

EXPERIMENTAL RESEARCHES ON SCROFULOUS AND TUBERCULOUS INFLAMMATION OF JOINTS. By M. Schüller. (*Centralblatt f. Chirurg.*, 1878, No. 43.

The starting point of the experiments was the well-known fact, that very slight injuries suffice to produce grave joint diseases in some individuals, while others will bear much more severe injuries without any such consequences. Healthy animals likewise, can sustain severe traumatic lesions of joints without more than mere temporary discomfort.

In order to produce a tuberculous tendency in animals, Schüller inoculated rabbits and dogs with tuberculous sputa, pieces of phthisical lungs or simple bacteria derived from tubercles and cultivated in nutrient solutions, according to the plan of Klebs. The fluids were injected into the lungs or inhaled by the animals from atomizers. In the course of some weeks, such animals become affected with pulmonary and even general miliary tuberculosis.

After the inoculation the animals were bruised so as to injure the knee-joint. Within some weeks the joints were examined, and in nearly all cases an inflammation was observed, consisting in a granular proliferation of the synovial membrane and a considerable swelling of the ends of the bones, which was noticeable during life as a tumefaction of the joint. In rare cases the joint suppurated.

Similar, although milder lesions were produced by direct injection of the infective fluids into the joint.

